# Screening of Wood Raw Materials for Low-Odor Fiberboard and Particleboard Production: Analysis and Evaluation Based on Volatile Odor Compounds

**DOI:** 10.3390/polym17172429

**Published:** 2025-09-08

**Authors:** Bo Liu, Fang Yang, Lina Tang, Xianwu Zou, Liming Zhu, Qian Chen, Bin Lv, Yuejin Fu

**Affiliations:** 1 Research Institute of Wood Industry, Chinese Academy of Forestry, Beijing 100091, China; liubo@caf.ac.cn (B.L.); 18211090798@163.com (L.T.); xwzou@caf.ac.cn (X.Z.); izhulm@caf.ac.cn (L.Z.); chenqian0610@126.com (Q.C.); zj3@caf.ac.cn (B.L.); 2 Zhongbei Guojian (Beijing) Testing Technology Co., Ltd., Beijing 100070, China; 15830951725@163.com

**Keywords:** wood, wood-based panels, Volatile Organic Compounds (VOCs), odor intensity, odor activity value, risk value

## Abstract

Woody raw materials of wood-based panels like fiberboard and particleboard are one of the primary sources of product odor and one of the indicators affecting the comprehensive health risk assessment of wood-based panel products. This study employed Gas Chromatography-Mass Spectrometry-Olfactometry (GC-MS-O) to investigate the odorant composition and odor characteristics, including Total Odor Concentration (TOC), odor intensity (OI), odor activity value (OAV), and risk value (RV), of 22 wood species commonly used in fiberboard and particleboard production in China. This research identified the major odor-active compounds in wood and provided recommendations for selecting wood raw materials suitable for low-odor fiberboards and particleboards produced by integrating RV and toxicity classification data. The results showed that the main compound types influencing wood odor in 22 wood species were predominantly terpenes, aldehydes, and alcohols. Woods of *Cinnamomum*, *Machilus*, and *Pinus* contained a higher number of dominant odor compounds (OAV > 1 and OI ≥ 3). Wood with stronger odor intensity included *Cinnamomum*, *Pinus*, *Machilus*, *Bischofia*, and *Saurauia*. The total RV of *Cinnamomum*, *Pinus*, *Machilus*, *Cunninghamia*, and *Bombax* wood exceeded one, necessitating special attention when used as raw materials for wood-based panels. Camphor in *Cinnamomum* and *Machilus* wood was the most concentrated odorant, followed by 3-Carene in *Pinus* wood. Odorants with high OAV included Longifolene, δ-Cadinene, Terpinen-4-ol, 2-Nonenal, γ-Terpinene, d-Limonene, 3-methyl-Butanal, Octanal, α-Pinene, Hexanal, D-Camphor, and trans-Calamenene. Odorants with high RV included terpenes, alcohols, aldehydes, and ketones, such as Camphor, 3-Carene, Eucalyptol, α-Terpineol, β-Pinene, α-Santalene, δ-Cadinene, Safrole, Longifolene, and d-Limonene. Focusing on the reduction and control of these odor-active compounds represents a primary approach to mitigating odors in fiberboard and particleboard products. However, addressing health risks associated with product odors requires additional attention to four specific substances: Safrole, Camphor, Eucalyptol, and α-Terpineol. Although the total RV for the five wood species exceeds one, this does not necessarily mean the final wood-based panel product’s RV exceeds one, as it also depends on the influence of the production process. Therefore, further research should be conducted to investigate the effects of various process parameters in wood-based panel production on the odor compounds present in the final panels. From a comprehensive perspective, considering the overall odor characteristics of wood volatiles, all 18 wood species (*Salix*, *Populus*, *Rhaphiolepis*, *Ligustrum*, *Prunus*, *Fagus*, *Pterocarya*, *Firmiana*, *Celtis*, *Cunninghamia*, *Bombax*, *Bischofia*, *Ficus*, *Saurauia*, *Eucalyptus*, *Aleurites*, *Melia*, *Bridelia*) are suitable for the production of low-odor fiberboards and particleboards.

## 1. Introduction

With the improvement of living standards and the enhancement of health awareness, indoor air quality (IAQ) has attracted increasing widespread attention. Wood-based panels, particularly particleboard and fiberboard, as core materials in home decoration and furniture manufacturing [1,2], release Volatile Organic Compounds (VOCs) and odors during use. These emissions have become key factors affecting indoor environmental comfort and consumers’ health perception [3,4,5]. Strong, unpleasant wood odors not only reduce the market acceptance of products but may also trigger negative psychological responses in consumers and even pose potential health risks [6,7,8,9,10]. Therefore, the development and production of “low-odor” wood-based panel products have become an important direction for the transformation and upgrading of the global panel industry [11].

Currently, the industry’s control over odors in wood-based panels mainly focuses on post-production processes, such as using low-odor adhesives, adding adsorbents, or applying post-treatment purification [12,13]. However, wood itself, as a complex biomass material, contains volatile odorous compounds that form an important source of the base odor of panels. This aspect is often overlooked during the raw material screening stage [14]. Significant differences exist in the composition, concentration, and odor characteristics of volatile compounds among different tree species and different parts of the wood [15]. If this key factor can be scientifically evaluated and controlled at the front end of production, i.e., during the wood raw material screening process, it will provide the most economical and effective approach to fundamentally reduce the odor intensity of wood-based panels and improve their odor quality.

Although studies on wood volatiles have been reported, most research has focused on single tree species or a limited number of extracts [16,17], lacking systematic comparative studies on the odorant profiles of wood species commonly used as industrial raw materials for wood-based panels. More importantly, traditional research largely relies on chemical quantitative analysis by Gas Chromatography-Mass Spectrometry (GC-MS) but often fails to effectively correlate chemical data with sensory odor characteristics and potential health risks, such as odor type, threshold odor concentration (TOC), the lowest concentration of interest (LCI), odor intensity (OI), and odor activity values (OAVs). This makes it difficult to directly apply such research to industrial raw material screening guidelines.

This study aims to fill this gap. A total of 22 wood species commonly used in China’s manufacturing of particleboard and fiberboard were systematically collected and analyzed. By combining the chemical analysis technique of Headspace Gas Chromatography-Mass Spectrometry (HS-GC-MS) with sensory evaluation (GC-Olfactometry) methods, the key volatile odor compounds in various woods and their calculated odor activity values (OAVs) to scientifically assess the contribution of each compound to the overall odor were accurately identified. Furthermore, this study introduced risk values for toxicological assessment of high-risk odor substances. The correlations between the mass concentration of VOCs, odor activity, and toxicity values (risk values) were established, and the impact of wood species selection on the odor compounds in particleboard and fiberboard was explored. Thereby, the standard for wood raw material screening was elevated from a singular focus on “odor intensity” to a comprehensive consideration of “odor safety”.

Fully utilizing forestry residues, secondary fuelwood, and artificial forests to produce wood-based panels is an important way to solve the problem of wood resource scarcity in China [18]. In recent years, due to the rise in the price of timber raw materials, in addition to common forestry residues, such as branch and shoot materials produced by forest logging, thinning, and branch materials during the nurturing process, and truncated and veneer materials produced during the timber production process, the branch materials of roadside trees that are regularly pruned and shaped every year have gradually become raw materials for the production of particleboard and fiberboard. The sources and types of odorants in particleboard and fiberboard will become more diverse and complex. It is particularly important to conduct a systematic analysis of the chemical composition, odor characteristics, and risk value (RV) of woody raw materials in order to understand the sources of odor substances, evaluate the problem of odors reasonably, and accurately reduce the odor of particleboard and fiberboard.

The main objectives of this study are to reveal the unique odor composition and odor characteristics of different wood raw materials, to identify advantageous wood species with inherently low-odor characteristics through comparative analysis, and to provide data support and theoretical reference for establishing a scientific wood raw material screening method based on volatile odor compound data. Ultimately, an “Odorant Database for Wood Raw Materials in Wood-Based Panels” established in this study will directly serve the industry, providing manufacturers with a precise decision-making basis when selecting wood raw materials. This will promote the development of low-odor, high-value-added wood-based panel products to meet the growing market demand for healthy and environmentally friendly materials.

## 2. Materials and Methods

### 2.1. Materials

Twelve species of wood logs were sourced from the raw material warehouse of the 200,000 m^3^ fiberboard production line in Zhenjiang, Jiangsu Daya Wood-based Panels Group, including *Cinnamomum camphora* (L.) Presl, *Pinus* sp., *Populus* sp., *Pterocarya stenoptera* C. DC, *Rhaphiolepis* sp., *Prunus* sp., *Fagus* sp., *Ligustrum* sp., *Firmiana* sp., *Melia* sp., *Salix* sp., and *Celtis* sp. Twelve species of wood logs were sourced from the raw material warehouse of the 400,000 m^3^ particleboard production line in Nanning, Guangxi Fenglin Wood Industry Group, including *Eucalyptus* sp., *Melia* sp., *Bombax* sp., *Cunninghamiae* sp., *Machilus* sp., *Bischofia* sp., *Saurauia* sp., *Celtis* sp., *Pinus* sp., *Aleurites* sp., *Ficus* sp., and *Bridelia* sp. The basic information on woody raw materials can be found in Supplementary Materials Tables S1 and S2.

Wood logs were sawn into 0.5 m segments, transported back to the laboratory via cold-chain logistics, and stored at −25 °C. Before experiments, the log segments were cut into 1 cm-thick discs and treated in a 60 °C oven for 70 min, to avoid the adverse effects of water on the injection port, liner, chromatographic column, mass spectrometer detector, and analytical results. These discs were then cut into 6 mm-wide small wood strips, which were further sliced into 1 mm-thick wood shavings.

### 2.2. Chemical Composition and Odor Characteristics Analysis of Odorants in Woods

A wood sample (1 g) was added to a 20 mL headspace bottle, which was then capped and sealed. After labeling, the samples were placed in the autosampler tray. Analysis was performed using a Tenax tube coupled with a dynamic headspace thermal desorption autosampler (Gerstel TD3.5+, Gerstel, Mülheim, Germany) and GC-MS (Agilent 8890/5977B, Agilent Technologies, Santa Clara, CA, USA). The detection procedure, GC-MS parameters, compound identification, quantitative analysis with the external standard method, and odor assessment tests all followed our laboratory-optimized methods, as detailed in Supplementary Table S3 and Ref. [14]. The TOC of odor substances in woody raw materials was determined through experiments, and combined with literature research [19], a TOC database for woody raw materials was established.

### 2.3. OAV and Risk Value Analysis of Odorants in Woods

The odor activity value (OAV) of the odor substance was calculated according to Formula (1) [20]. The exposure limit index (LCI) of the odor substance was collected from the EU Report No. 29 [21], the German Federal Environment Agency (UBA) “AGBB Evaluation Scheme 2015” report [22], and data published on the official website of the US Environmental Protection Agency [23]. The RV of this odorant was then calculated using Equation (2) for risk assessment specified in the BS EN 16516:2017 standard [24].

(1) $$OAV=\frac{C}{OT}$$

where

*OAV*—the odor activity value, non-dimensional;

*C*—the mass concentration of odor compound, mg/m^3^;

*OT*—the olfactory threshold of odor compound, mg/m^3^.

(2) $$RV=\frac{C}{LCI}$$

where

RV—the risk value, non-dimensional;

C—the mass concentration of the odor compound, mg/m^3^;

LCI—the lowest concentration of the interest value of the odor compound, mg/m^3^.

## 3. Results and Discussion

### 3.1. Chemical Components of Odorants in Woods

**Figure 1:**
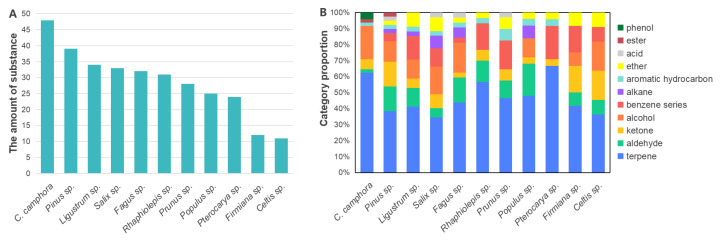

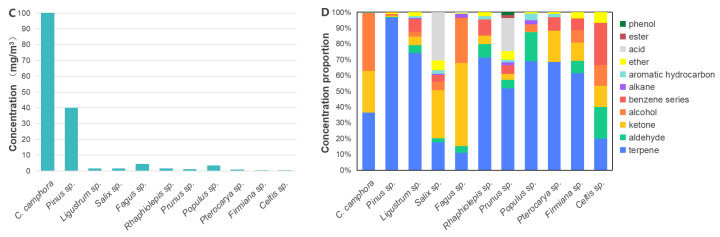
Quantity and concentration of odor substances in fiberboard woody raw materials. (**A**) Quantity histogram of odor substances in different woods; (**B**) Quantity proportion of compound categories in different woods; (**C**) Histogram of total concentration of odor substances in different woods; (**D**) Concentration proportion of odor substances in different compound categories.

**Figure 2:**
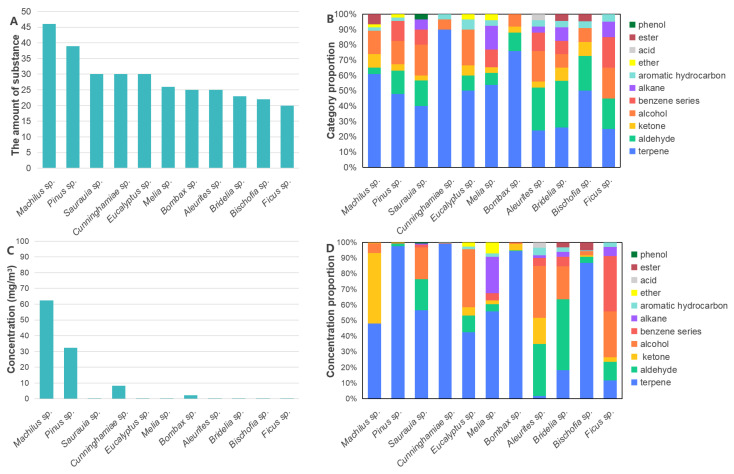
Quantity and concentration of odor substances in particleboard woody raw materials. (**A**) Quantity histogram of odor substances in different woods; (**B**) Quantity proportion of compound categories in different woods; (**C**) Histogram of total concentration of odor substances in different woods; (**D**) Concentration proportion of odor substances in different compound categories.

The Total Ion Chromatograms (TICs) of VOCs from 22 wood raw materials for fiberboard and particleboard are shown in Supplementary Figures S1 and S2. The quantity, types, concentration, and composition of volatile substances are presented in Figure 1 and Figure 2. Substances released from the woods included terpenes, aldehydes, ketones, alcohols, BTEX compounds (benzene series), alkanes, aromatic hydrocarbons, ethers, acids, esters, and phenols. However, the composition, diversity, concentration levels, and proportions of these substances varied significantly among different wood species.

Regarding the number of odorants, as shown in Figure 1A and Figure 2A, and Table 1 and Table 2, *Cinnamomum camphora* wood released the greatest diversity of substances (48) among fiberboard woody raw materials, while *Firmiana* (12) and *Celtis* (11) woods released the fewest. Other woods released between 20 and 40 substances. Among particleboard woody raw materials, *Machilus* wood released the most diverse volatile substances (46), followed by *Pinus* wood (39), with other woods releasing between 20 and 30 substances. The higher number of volatile substances released by *C. camphora* and *Machilus* woods is attributed to both belonging to the Lauraceae family. A distinctive feature of Lauraceae woods is the presence of abundant oil cells. These oil cells contain rich reserves of Volatile Organic Compounds with preservative properties, which serve as the primary odor-emitting functional groups [25].

Regarding the compositional proportions of different compound groups, as shown in Figure 1B and Figure 2B, among fiberboard woody raw materials, *Pinus* wood contained the most diverse chemical categories (10 types, excluding phenols). *Pterocarya* wood contained the least diverse chemical categories (composed of only five types: terpenes, ketones, BTEX compounds, aromatic hydrocarbons, and ethers). Across all fiberboard woods, terpenes constituted the largest proportion of organic compounds (36–67%), followed by alcohols (8–21%), and then aldehydes (2–20%). Among particleboard woody raw materials, *Aleurites* and *Bridelia* woods contained the most diverse chemical categories (eight types each). *Cunninghamia* wood contained the least diverse chemical categories (composed of only three types: terpenes, alcohols, and aromatic hydrocarbons). Across all particleboard woods, terpenes constituted the largest proportion of organic compounds (ranging from 24% to 90%), followed by aldehydes (4–30%), and then alcohols (8–23%). In woods, terpenoid compounds primarily originate from extractives. In wood-based panels, terpenoid compounds mainly derive from the wood itself [26,27]. Therefore, the Chinese National Standard GB/T 44690-2024 “Classification of volatile organic compounds emission from wood-based panels and their products”, implemented on 1 April 2025, designated nine naturally occurring terpenoid Volatile Organic Compounds emitted by woods as exempted compounds in the testing of VOC emissions from wood-based panels and their products [28]. These compounds are as follows: α-Pinene, β-Pinene, 3-Carene, d-Limonene, Phellandrene, Caryophyllene, 2-Camphanone (Camphor), 2-Camphanol (Borneol), and Eucalyptol. Acetic acid, Hexanal, and other small molecules were primarily derived from the degradation products of cellulose and hemicellulose in wood [25,29].

Regarding the total Volatile Organic Compound (VOC) concentrations across different wood species, as shown in Figure 1C and Figure 2C, among fiberboard woody raw materials, *C. camphora* and *Pinus* woods exhibited the highest total VOC concentrations at 101.40 mg/m^3^ and 39.03 mg/m^3^, respectively. All other woods had concentrations below 1 mg/m^3^. Among particleboard woody raw materials, the top four woods by total VOC concentration were *Machilus* (62.32 mg/m^3^), *Pinus* (32.23 mg/m^3^), *Cunninghamia* (8.38 mg/m^3^), and *Bombax* (2.19 mg/m^3^). All other woods had total VOC concentrations below 1 mg/m^3^.

Regarding the concentration proportions of different compound categories, as shown in Figure 1D and Figure 2D, in fiberboard woody raw materials, the concentration proportion of terpenes exceeded 50% of total VOC concentration in all woods except for *Fagus* (11%), *Salix* (18%), *Celtis* (20%), and *C. camphora* (36%). *Pinus* wood showed the highest terpene proportion at 97%. Fagus wood had the highest concentration proportion of ketones (52%), *C. camphora* wood had the highest concentration proportion of alcohols (37%), *Salix* wood had the highest concentration proportion of acids (30%), and *Celtis* wood had the highest concentration proportion of benzene derivatives (27%). In particleboard woody raw materials, the concentration of terpenes in other woods exceeded 40% in all woods except for *Aleurites*, *Ficus*, and *Bridelia*. Among them, the concentration proportion of terpenes in *Pinus*, *Cunninghamiae*, *Bombax*, and *Bischofia* wood was as high as 98% to 87%.

Detailed data on total odorant concentrations and compound category proportions for individual wood species were provided in Supplementary Tables S4–S7 (fiberboard) and Tables S8–S11 (particleboard).

### 3.2. Odor Characteristics of Odorants in Woods

#### 3.2.1. Odor Intensity of Odorants in Woods

Odor intensity (OI) refers to numerical or descriptive representations of odor strength. Grading systems for odor intensity varied across countries and regions. Both ASTM E544 [30] and EN 13725 [31] were authoritative standard methods—the former focused on subjective intensity matching, while the latter specialized in threshold determination. In contrast, ISO 16820 [32] adopted a more intuitive categorical scale, making it better suited for general applications. Countries like Japan and China used this categorical scale system, specifically a 6-level intensity classification:

Level 0: No odor (odorless);

Level 1: Barely perceptible (very weak and ambiguous);

Level 2: Clearly perceptible but low intensity;

Level 3: Distinct, moderate-intensity odor;

Level 4: Strong odor (cannot be ignored);

Level 5: Very strong odor (uncomfortable or intolerable).

In this study, odorants with higher odor intensity and greater odor activity values are listed in Table 1 and Table 2. Among the 22 wood species, there were a total of 28 odor-active compounds with an odor intensity higher than Level 3. Terpenes are the most abundant, followed by aldehydes, alcohols, ketones, esters, and ethers. In wood species for fiberboard raw materials, *Cinnamomum* and *Pinus* woods contained the most odorants with distinct odor intensity (OI ≥ 3), 8 and 4, respectively. In wood species for particleboard raw materials, *Machilus* and *Pinus* woods contained the most odorants with high odor intensity (OI ≥ 3), 4 and 6, respectively. The other 18 wood species can be used as raw materials for low-odor fiberboard and particleboard in terms of odor intensity.

The compounds with the highest concentration, OI, OAV, and RV in wood species for fiberboard and particleboard raw materials are listed in Table 3 and Table 4. The highest OI (Level 5) was 3-Carene in *Pinus*. Compounds with Level 4 odor intensity included Safrole in *Machilus* wood, 3-Carene, Camphor, and Hexanal in *Pinus* wood, trans-α-Bergamotene in *Bombax* wood, as well as 2-Methyl-Butanal and trans-Sabinene hydrate in *Saurauia* wood (Total: seven compounds).

Volatile compounds with high odor intensity typically possess the following characteristics: very low odor thresholds, specific molecular structures (functional groups and shape), relatively high vapor pressure, and high affinity for olfactory receptors. They also often interact with other substances, exhibiting either synergistic effects or masking effects. Therefore, in the case of wood as a complex mixture of volatile compounds, its assessment requires integrated consideration alongside other odor characteristics.

#### 3.2.2. Odor Activity Value of Odorants in Woods

The odor activity value (OAV) is a key metric for evaluating the contribution of a volatile compound to the overall odor profile. It is generally recognized that an OAV greater than one indicates that the compound’s concentration exceeds its odor threshold, signifying a significant contribution to the overall odor and classifying it as a critical odorant [33].

As shown in Table 1 and Table 2, *Cinnamomum* and *Pinus* woods also exhibited the highest number of key odorants (OAV > 1), 35 and 17, respectively. *Machilus* and *Pinus* woods also showed the highest number of key odorants (OAV > 1), 19 and 15, respectively. A higher OAV value indicates that the substance contributes more significantly to the overall odor and is more likely to be a “key odor compound” or an “off-odor compound”. The OAV itself is an indicator in sensory science rather than a direct measure of health or safety. It serves as an early warning and directional signal; that is, a high OAV value acts as a strong indicator. When the OAV of a substance is significantly greater than one, it suggests that the odor of this substance is particularly prominent and intense. The OAV highlights potential risks. Many VOCs harmful to health, such as formaldehyde, benzene, toluene, and xylene, inherently possess strong irritating odors and typically exhibit low odor thresholds. Therefore, wood-based panels manufactured from these species (*Cinnamomum*, *Pinus*, *Machilus*) were likely to exhibit stronger odor profiles than those made from other wood species. From the perspective of OAVs, it is not recommended to use them as raw materials for low-odor wood-based panels, or they should be added in limited quantities based on specific panel requirements.

As shown in Table 3 and Table 4, the highest OAV was Terpinen-4-ol in *Cinnamomum* (OAV = 981.82) in wood c, and Longifolene in *Pinus* (OAV = 2253.99) in wood species for particleboard raw materials. There were also many other odorants with a high OAV, such as δ-Cardine in *Bombax* (OAV = 1210.13), 2-Nonenal in *Bischofia* (OAV = 499.59), γ-Terpinene in *Machilus* (OAV = 197.14), d-Limonene in *Pinus* (OAV = 118.86), 3-methyl-Butanal in *Populus* (OAV = 77.87), 3-methyl-Butanal in *Saurauia* (OAV = 76.09), Octanal in *Bridelia* (OAV = 38.87), and α-Pinene in Ligustrum (OAV = 28.78). A high OAV value may indicate poor indoor air quality and potential exposure risks to harmful substances, necessitating further health risk assessments. However, it is important to clearly distinguish the following concepts: a high OAV does not equate to high toxicity. OAV measures how strong and pronounced the odor of a substance is, while health risks depend on toxicity and exposure dosage.

From Table 3 and Table 4, it can also be observed that the following eight compounds were most frequently identified in key odor metrics among the 22 wood species used for fiberboard and particleboard. They were α-Pinene, 3-Carene, Hexanal, δ-Cadinene, 3-methyl-Butanal, Ethanol, d-Limonene, and Eucalyptol.

### 3.3. Risk Value of Odorants in Woods

Whether a substance poses a health hazard depends on its inherent toxicity (such as carcinogenicity and teratogenicity) as well as the concentration and duration of human exposure to it. Acetic acid (vinegar acid): It has a strong sour odor and a very low olfactory threshold. In vinegar, its OAV is high, but it is essentially harmless to humans. Formaldehyde: It has both an irritating odor (with a relatively low olfactory threshold) and is a recognized carcinogen. When its OAV is high, it typically indicates a high health risk. Carbon monoxide (CO): This is an extreme counterexample. Carbon monoxide is a colorless, odorless, and lethal toxic gas. Its olfactory threshold can be considered infinitely high. Therefore, its OAV is always zero, yet even very low concentrations of CO can lead to poisoning and death. This demonstrates that relying solely on odor to assess safety risks is extremely dangerous. Thus, this study introduces RV.

The relationship between OAV and OI can be summarized as follows: OAV is a necessary but insufficient condition for OI, and the two are generally positively correlated, though not in a simple linear manner. In complex mixtures, this relationship becomes even more intricate. Therefore, by screening odor-active compounds based on their significant odor impact (OAV ≥ 1 and OI ≥ 3) (Table 5), we can clearly focus on the RV values and toxicity of these compounds. Table 5 showed that there were 16 major odor-active compounds, primarily terpenes and a few aldehydes and ketones. These mainly originated from six wood species: *Cinnamomum*, *Pinus*, *Machilus*, *Populus*, *Rhaphiolepis*, and *Eucalyptus*. With the exception of α-Santalene, 3-Carene, and Safrole, the major odor-active compounds exhibited very low RV. α-Santalene and 3-Carene are natural terpenes present in wood. Although their RV exceed one, their toxicity levels remain low, consistent with the other major odor-active compounds. Safrole, however, is an orally carcinogenic substance that poses risks of toxicity through skin contact and inhalation. Corresponding control measures need to be taken during the production of wood-based panels.

Meanwhile, odor compounds with RV ≥ 1 found in wood are listed in Table 6. A total of 15 odor compounds with RV values exceeding one were identified, originating from five wood species: *Cinnamomum*, *Pinus*, *Machilus*, *Cunninghamia*, and *Bombax*. Among these, aside from Safrole, which requires special control measures, Camphor, Eucalyptol, and α-Terpineol are classified as moderately toxic. These compounds primarily derive from *Cinnamomum* and *Machilus* woods, both belonging to the Lauraceae family. Therefore, it is recommended to avoid using Lauraceae species as raw materials in the production of low-odor or environmentally friendly wood-based panels whenever possible. The remaining 11 odor compounds with RV ≥ 1 were predominantly terpenes, all of which exhibited low toxicity levels.

China’s Technical Guideline for Environmental Impact Assessment—Ambient Air (HJ 2.2-2018) [34] explicitly stipulated that non-carcinogenic health risk assessments must be conducted for characteristic pollutants (including odorants). According to the AGBB Evaluation Scheme (2015) issued by the German Federal Environment Agency (UBA), the sum of all risk values (∑RV) for hazardous substance exposure risk assessment in the construction industry must not exceed the value of one in the VOC emission assessment procedure for building products [22].

The number of compounds with an RV greater than one for odor substances in 22 wood species and the sum of all risk values (∑RV) are shown in Table 1 and Table 2. In wood species for fiberboard raw materials, *Cinnamomum* and *Pinus* woods contained the highest number of odorants with RV ≥ 1 (8 and 3 compounds, respectively). This resulted in ∑RV values significantly exceeding one for both species. In wood species for particleboard raw materials, four species exhibited odorants with RV ≥ 1, including *Machilus*, *Pinus*, *Cunninghamia*, and *Bombax*. These same four species also showed ∑RV > 1.

**Figure 3:**
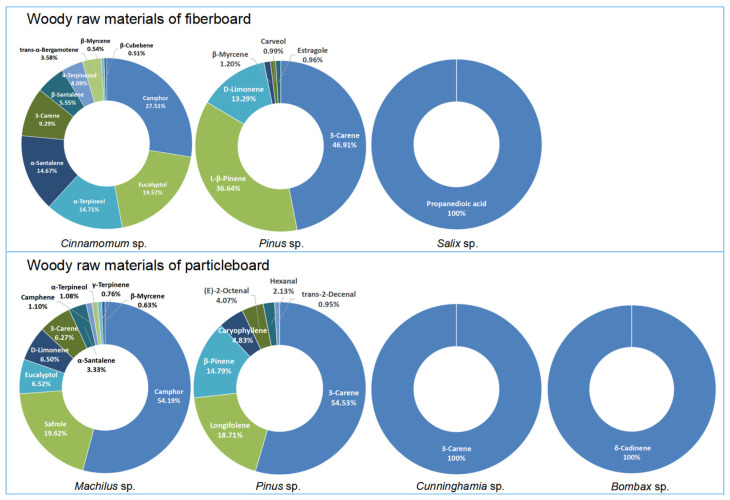
Disk chart of the RV proportion of odorants with RV > 0.2 in woods.

Figure 3 shows the proportion of RVs for odorants with RV > 0.2 across the 22 wood species. Camphor constituted the largest RV proportion in *Cinnamomum* and *Machilus*. 3-Carene represented the highest RV proportion in *Pinus* and *Cunninghamia*. Propanedioic acid and δ-Cadinene were the dominant RV contributors in *Salix* and *Bombax*. 3-Carene and δ-Cadinene exhibited very low inhalation toxicity. The risk assessment for inhalation exposure to compounds such as Camphor, Eucalyptol, and α-Terpineol is primarily based on their irritant effects.

RV serves as a crucial “signal light” system. It translates complex toxicological data into an easily understandable numerical value, assisting decision-makers, engineers, and public health experts in prioritizing the most pressing environmental health issues and formulating corresponding control standards. When multiple odorants with similar mechanisms of toxicity coexist, a sum of risk values (∑RV) exceeding one (typically referring to non-carcinogenic risk) indicates potential health hazards. Therefore, special attention is required when these wood species are used as raw materials for wood-based panels.

Crucially, during wood-based panel manufacturing processes, including raw material screening, washing, drying, mat forming, hot pressing, and cooling after pressing, the concentrations of volatile odorants are likely to be significantly reduced due to water leaching, airflow exposure, and temperature elevation. Consequently, both the OAV and RV decreased substantially. It is noted that wood odor profiles do not fully equate to final panel odors, as the latter result from the cumulative effects of wood raw materials, adhesives, waterproofing agents, lubricants, release agents, and other auxiliary materials throughout the production chain.

## 4. Conclusions

Through detection and literature retrieval, the concentrations of odor compounds, predominant odorants, and odor activity values of 22 woody raw materials for fiberboard and particleboard in China were quantitatively determined. A database was established for the TOC, LCI, OI, OAV, and RV of odor substances in these woods. More importantly, by adopting the LCI inventory and introducing the RV as an evaluation criterion, this approach ensured a comprehensive evaluation.

The results indicated that the primary compounds affecting wood odor across the 22 wood species were predominantly terpenes, aldehydes, and alcohols. Woods of *Cinnamomum*, *Machilus*, and *Pinus* exhibited a greater number of dominant odor compounds (OAV > 1). Species with higher odor intensity include *Cinnamomum*, *Pinus*, *Machilus*, *Bischofia*, and *Saurauia*. Notably, woods such as *Cinnamomum*, *Pinus*, *Machilus*, *Cunninghamia*, and *Bombax* showed a total RV exceeding one, necessitating special attention when used as raw materials for wood-based panels.

Twelve odor compounds with a high OAV and ten with elevated RVs, including terpenes, alcohols, aldehydes, and ketones, were identified as the predominant odorants affecting the 22 wood species. Broad-spectrum coverage or targeted capture of these compounds represented the primary approach for reducing and controlling odors in wood-based panel products. Although the total RVs exceeded one for five wood species, this does not necessarily imply that the final panel products will exhibit RV > 1, as outcomes depended critically on the influence of production processes. Therefore, further research should investigate the individual and cumulative effects of processing stages, raw materials, and additives on the odor profiles of finished wood-based panels.

## Figures and Tables

**Table 1:** Quantity of odorants with higher odor intensity, odor activity value, and risk value in fiberboard woody raw materials.

Wood Name	Total Number of Odorants	OI		OAV > 1	RV ≥ 1	Σ RV
		OI ≥ 1	OI ≥ 3			
*Cinnamomum* sp.	48	15	8	35	8	67.47
*Pinus* sp.	39	9	4	17	3	26.58
*Salix* sp.	33	3	0	2	0	0.20
*Populus* sp.	25	2	1	9	0	0.19
*Rhaphiolepis* sp.	31	4	1	5	0	0.10
*Ligustrum* sp.	34	0	0	3	0	0.09
*Prunus* sp.	28	3	0	4	0	0.08
*Fagus* sp.	32	5	2	6	0	0.06
*Pterocarya* sp.	24	3	0	2	0	0.05
*Firmiana* sp.	12	2	0	1	0	0.01
*Celtis* sp.	11	0	0	1	0	0.01

Notes: OI—odor intensity; OAV—odor activity value; RV—risk value; and Σ RV—the sum of all compounds RV.

**Table 2:** Quantity of odorants with higher odor intensity, odor activity value, and risk value in particleboard woody raw materials.

Wood Name	Total Number of Odorants	OI ≥ 1		OAV > 1	RV ≥ 1	Σ RV
		OI ≥ 1	OI ≥ 3			
*Machilus* sp.	46	6	4	19	6	35.73
*Pinus* sp.	39	10	6	15	4	22.99
*Cunninghamia* sp.	30	2	0	7	1	5.60
*Bombax* sp.	25	2	0	5	1	1.38
*Bischofia* sp.	22	3	0	6	0	0.48
*Ficus* sp.	20	0	0	0	0	0.19
*Saurauia* sp.	30	5	0	5	0	0.13
*Eucalyptus* sp.	30	4	1	7	0	0.09
*Aleurites* sp.	25	1	1	4	0	0.04
*Melia* sp.	26	2	0	2	0	0.03
*Bridelia* sp.	23	0	0	5	0	0.02

Notes: OI—odor intensity; OAV—odor activity value; RV—risk value; and Σ RV—the sum of all compounds RV.

**Table 3:** The maximum value of odor characteristics and risk values of woody raw materials for fiberboard.

Wood Name	C		OI		OAV		RV	
	C_max_	mg/m^3^	OI_max_	Level	OAV_max_	Value	RV_max_	Value
*Cinnamomum* sp.	Camphor	26.44	Caryophyllene, α-Santalene, cis--Farnesene, β-Sesquiphellandrene, β-Cubebene, γ-Selinene, Citronellol, α-Thujene	3	Terpinen-4-ol	981.82	Camphor	17.63
*Pinus* sp.	3-Carene	18.29	3-Carene	5	d-Limonene	118.86	3-Carene	12.19
*Salix* sp.	Acetoin	0.05	Estragole, o-Cymene	2	Hexanal	2.92	Propanedioic acid	0.15
*Populus* sp.	Copaene	0.12	3-methyl-Butanal	3	3-methyl-Butanal	77.87	Copaene	0.08
*Rhaphiolepis* sp.	α-Pinene	0.03	D-Camphor	3	D-Camphor	4.18	α-Pinene	0.01
*Ligustrum* sp.	α-Pinene	0.09	Hexanal	1	α-Pinene	28.78	α-Pinene	0.03
*Prunus* sp.	α-Pinene	0.03	α-Pinene	2	3-methyl-Butanal	4.53	α-Pinene	0.02
*Fagus* sp.	Acetoin	0.24	(R)-(-)-Leucinol	3	3-methyl-Butanal	10.90	Hexanal	0.01
*Pterocarya* sp.	α-Pinene	0.02	Camphene	3	trans-Calamenene	7.38	α-Pinene	0.01
*Firmiana* sp.	α-Pinene	0.01	Eucalyptol	2	Hexanal	1.47	α-Pinene	0.004
*Celtis* sp.	Benzene	0.004	d-Limonene, Estragole	1	Hexanal	2.34	Hexanal	0.003

Notes: C—concentration of odorants; OI—odor intensity; OAV—odor activity value; and RV—risk value.

**Table 4:** The maximum value of odor characteristics and risk values of woody raw materials for particleboard.

Wood Name	C		OI		OAV		RV	
	C_max_	mg/m^3^	OI_max_	Level	OAV_max_	Value	RV_max_	Value
*Machilus* sp.	Camphor	28.04	Safrole	4	γ-Terpinene	197.14	Camphor	18.69
*Pinus* sp.	3-Carene	17.87	3-Carene, Camphor, Hexanal	4	Longifolene	2253.99	3-Carene	11.91
*Cunninghamia* sp.	3-Carene	7.96	3-Carene, trans-Sabinene hydrate	2	δ-Cadinene	9.01	3-Carene	5.31
*Bombax* sp.	δ-Cadinene	1.94	trans-α-Bergamotene	2	δ-Cadinene	1210.13	δ-Cadinene	1.29
*Bischofia* sp.	3-Carene	0.28	2-Nonanone	4	2-Nonenal	449.59	2-Nonenal	0.20
*Ficus* sp.	Ethanol	0.01	—	0	Decanal	0.80	Decanal	0.002
*Saurauia* sp.	3-Carene	0.32	2-Methyl-Butanal, trans-Sabinene hydrate	4	3-methyl-Butanal	76.09	α-Pinene	0.05
*Eucalyptus* sp.	Ethanol	0.04	δ-Cadinene	3	Hexanal	12.39	3-methyl-1-Butanol	0.02
*Aleurites* sp.	Ethanol	0.02	Ethanol	3	D-Camphor	12.03	Hexanal	0.01
*Melia* sp.	3-Carene	0.004	2-methyl-Tetradecane	2	δ-Cadinene	2.08	3-Carene, Styrene	0.003
*Bridelia* sp.	Ethanol	0.01	Propanoic acid	1	Octanal	38.87	Decanal	0.01

Notes: C—concentration of odorants; OI—odor intensity; OAV—odor activity value; and RV—risk value.

**Table 5:** Odor characteristics of volatile odorants with OAV ≥ 1 and OI ≥ 3 in wood.

Wood Name	Odor Compound	CAS#	Odorant Type	OAV	OI	RV	Toxicity
*Cinnamomum* sp.	Caryophyllene	87-44-5	Light lilac scent	55.46	3	0.16	Low
	γ-Selinene	515-17-3	Citrus aroma	3.29	3	0.09	Low
	β-Sesquiphellandrene	20307-83-9	Herbaceous, fruit, wood aroma	2.89	3	0.12	Low
	cis-β-Farnesene	28973-97-9	Lemon, citrus	2.42	3	0.06	Low
	α-Santalene	512-61-8	Similar to cedar aroma	2.29	3	9.40	Low
	Citronellol	106-22-9	Rose fragrance	1.86	3	0.01	Low
	α-Thujene	2867-05-2	Wood scent	1.63	3	0.19	Low
*Pinus* sp.	Hexanal	66-25-1	Stimulating green grass and apples	92.02	4	0.11	Low
	Humulene	6753-98-6	Light lilac	53.40	3	0.06	Low
	3-Carene	13466-78-9	China fir odor	16.65	5	12.19	Low
*Machilus* sp.	Safrole	94-59-7	Cinnamomum camphora fragrance	129.50	4	6.77	Oral carcinogenicity
	1, 3-Benzodioxole, 4-methoxy-6-(2-propenyl)-	607-91-0	Acridity scent	100.02	3	—	Low
	3-Carene	13466-78-9	China fir odor	2.95	3	2.17	Low
	Linalyl acetate	115-95-7	Citrus aroma	1.03	3	0.03	Low
*Populus* sp.	Butanal, 3-methyl-	590-86-3	Ethereal, chocolate and peach aroma	77.86	3	0.003	Low
*Rhaphiolepis* sp.	D-Camphor	464-49-3	Camphoraceous, herb, wood scent	4.18	3	0.003	Moderate
*Eucalyptus* sp.	δ-Cadinene	483-76-1	Thyme herb aroma	5.27	3	0.01	Low

**Table 6:** Odor characteristics of volatile odorants with RV ≥ 1 in wood.

Wood Name	Odor Compound	CAS#	Odor Type	OAV	OI	RV	Toxicity Grade
*Cinnamomum* sp.	Camphor	76-22-2	Camphor scent	9.31	0	17.63	Moderate
	Eucalyptol	470-82-6	Camphor scent and refreshing herbal taste	1.88	0	12.54	Moderate
	α-Terpineol	98-55-5	Lilac aroma	36.67	0	9.42	Moderate
	α-Santalene	512-61-8	Similar to cedar aroma	2.29	3	9.40	Low
	3-Carene	13466-78-9	China fir odor	8.12	0	5.95	Low
	β-Santalene	511-59-1	Similar to cedar aroma	2.85	2	3.56	Low
	4-Terpinenol	562-74-3	Pepper aroma, light earthy scent, and aged wood aroma	981.82	0	2.62	Low
	trans-α-Bergamotene	13474-59-4	Lemon aroma	6.75	2	2.29	Low
*Pinus* sp.	L-β-Pinene	18172-67-3	Pine resin aroma	7.14	1	9.52	Low
	D-Limonene	5989-27-5	Lemon and orange	118.86	0	3.45	Low
	3-Carene	13466-78-9	China fir odor	16.27	4	11.91	Low
	Longifolene	475-20-7	Wood and iris-like scent	2553.99	2	4.09	Low
	β-Pinene	127-91-3	Pine resin aroma	0.26	0	3.23	Low
	Caryophyllene	87-44-5	Light lilac scent	368.03	2	1.06	Low
*Machilus* sp.	Camphor	76-22-2	Camphor scent	9.87	1	18.69	Moderate
	Safrole	94-59-7	Cinnamomum camphora fragrance	129.45	4	6.77	Oral carcinogenicity
	Eucalyptol	470-82-6	Camphor scent and refreshing herbal	0.34	0	2.25	Moderate
	D-Limonene	5989-27-5	Lemon and orange	77.08	0	2.25	Low
	3-Carene	13466-78-9	China fir odor	2.95	3	2.16	Low
	α-Santalene	512-61-8	Similar to cedar aroma	0.28	0	1.15	Low
*Cunninghamia* sp.	3-Carene	13466-78-9	China fir odor	7.28	2	5.31	Low
*Bombax* sp.	δ-Cadinene	483-76-1	Thyme herb fragrance	1210.13	1	1.29	Low

## Data Availability

The raw data supporting the conclusions of this article will be made available by the authors on request.

## Supplementary Materials

The following supporting information can be downloaded at: https://www.mdpi.com/article/10.3390/polym17172429/s1, Table S1. Basic information of wood species for fiberboard raw materials; Table S2. Basic information of wood species for particleboard raw materials; Figure S1. Total Ion Chromatograms of 11 wood species of fiberboard raw materials; Figure S2. Total Ion Chromatograms of 11 wood species of particleboard raw materials; Table S3. Methods of chemical composition and odor characteristic analysis of odorants in woods; Table S4. Quantity of compounds in different wood species of fiberboard raw materials; Table S5. Quantity proportion of compound categories in different wood species of fiberboard raw materials; Table S6. Total concentration of odor substances in different wood species for fiberboard raw materials; Table S7. Concentration proportion of odor substances in different compound categories of wood species for fiberboard raw materials; Table S8. Quantity of compounds in different wood species for particleboard raw materials; Table S9. Quantity proportion of compound categories in different wood species for particleboard raw materials; Table S10. Total concentration of odor substances in different wood species for particleboard raw materials; and Table S11. Concentration proportion of odor substances in different compound categories of wood species for particleboard raw materials.
