# Supplementary material for: Screening of Wood Raw Materials for Low-Odor Fiberboard and Particleboard Production: Analysis and Evaluation Based on Volatile Odor Compounds
Source: Polymers (Basel). 2025 Sep 8;17(17):2429. doi: 10.3390/polym17172429 (PMC12431084; doi:10.3390/polym17172429)
Supplement: Supplementary file 1 [file polymers-17-02429-s001.zip › polymers-3757705-supplementary.pdf]

Table S1. Basic information of wood species for fiberboard raw materials

Wood species	Family	Category	Common usage
Cinnamomum sp.	Lauraceae	Large tree	Landscape, Construction, Building, Furmiture, Vehicle, Wood-based panels, Chinese medicine
Pinus sp.	Pinuseae	Large tree	Garden, Construction, Furmiture, Wood-based panels
Populus sp.	Salicaceae	Large tree	Building, Furmiture, Interior decoration, Building templates, Base material of door, Wood-based panels
Pterocarya sp.	Juglandaceae	Large tree	Riverside tree, Garden, Roadside tree, Landscape tree, Papermaking, Furmiture, Wood-based panels, Matchstick, Cutting board, Chinese medicine
Rhaphiolepis sp.	Rosaceae	Shrub	View, Garden, Vessels, Chinese medicine
Prunus sp.	Rosaceae	Large tree	Furniture, Door, Wall panel, Toy, Musical instrument, Wood-based panels
Fagus sp.	Ulmaceae	Large tree	Furniture, Construction, Shipbuilding, Park and Landscape, Urban greening, Decoration
Ligustrum sp.	Oleaceae	Small tree or Shrub	Garden, Roadside tree, Landscape tree
Firmiana sp.	Moraceae	Large tree	Garden, Roadside tree, Landscape, Chinese medicine
Salix sp.	Salicaceae	Large tree	Furniture, Timber, Construction, Tool
Celtis sp.	Ulmaceae	Small tree	Afforestation, Landscape, Furniture, Box, Carving, Agricultural tool

Table S2. Basic information of wood species for particleboard raw materials

Wood species	Family	Category	Common usage
Machilus sp.	Lauraceae	Large tree	Garden, Construction, Furmiture, Wood-based panels
Pinus sp.	Pinuseae	Large tree	Garden, Construction, Furmiture, Wood-based panels
Bombax sp.	Bombacaceae	Large tree	Garden, Roadside tree, Box board, Matchstick, Papermaking,
Bischofia sp.	Euphorbiaceae	Large tree	Roadside tree, Riverside tree, Shade tree, Vessel, Furniture, Flooring
Aleurites sp.	Euphorbiaceae	Middle-sized tree	Street tree, Box board, Matchstick
Saurauia sp.	Actinidiaceae	Small tree	Chinese Medicine
Eucalyptus sp.	Myrtaceae	Large tree	Wood-based panels, Papermaking, Packaging, Construction, Mine pillars, Furmiture, Garden, Roadside tree
Bridelia sp.	Phyllanthaceae	Small tree	Architecture, Furniture, Vehicle, Agricultural tool, Utensil
Ficus sp.	Moraceae	Large tree	Garden, Shade tree, Roadside tree, Furniture, Handicrafts, Carving
Melia sp.	Meliaceae	Middle-sized tree	Garden, Architecture, Furniture, Wood-based panels
Cunninghamia sp.	Cupressaceae	Large tree, Megaphanerophyte	Wood-based panels, Furniture, Construction, Garden, Roadside tree

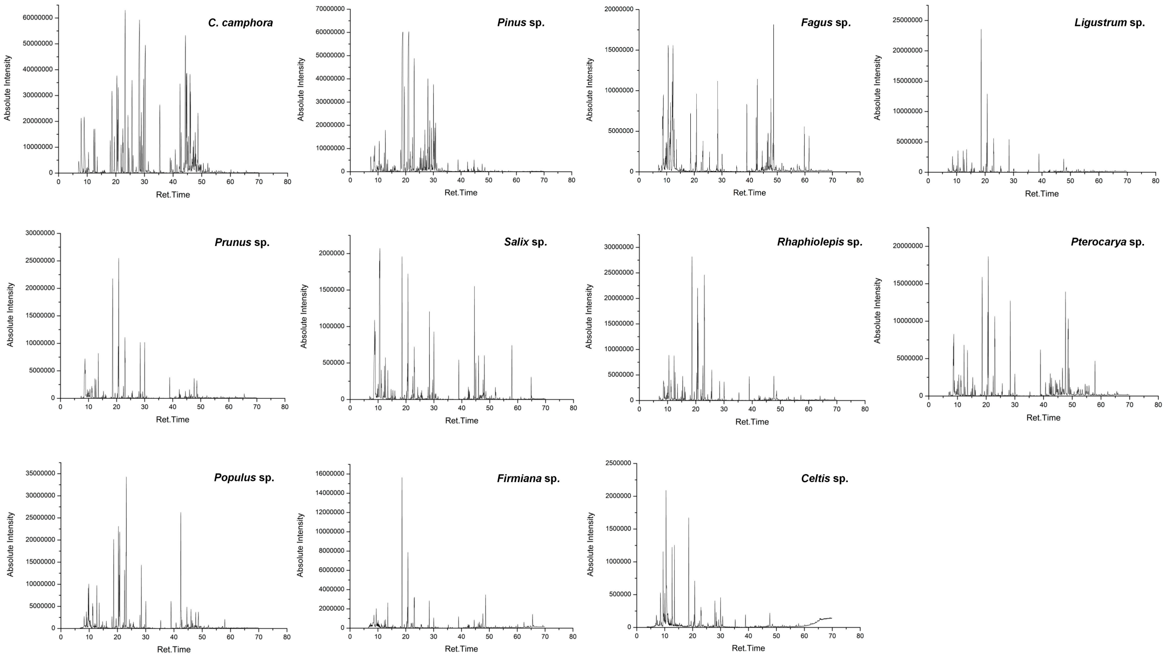

Figure S1. Total Ion Chromatograms of 11 wood species of fiberboard raw materials

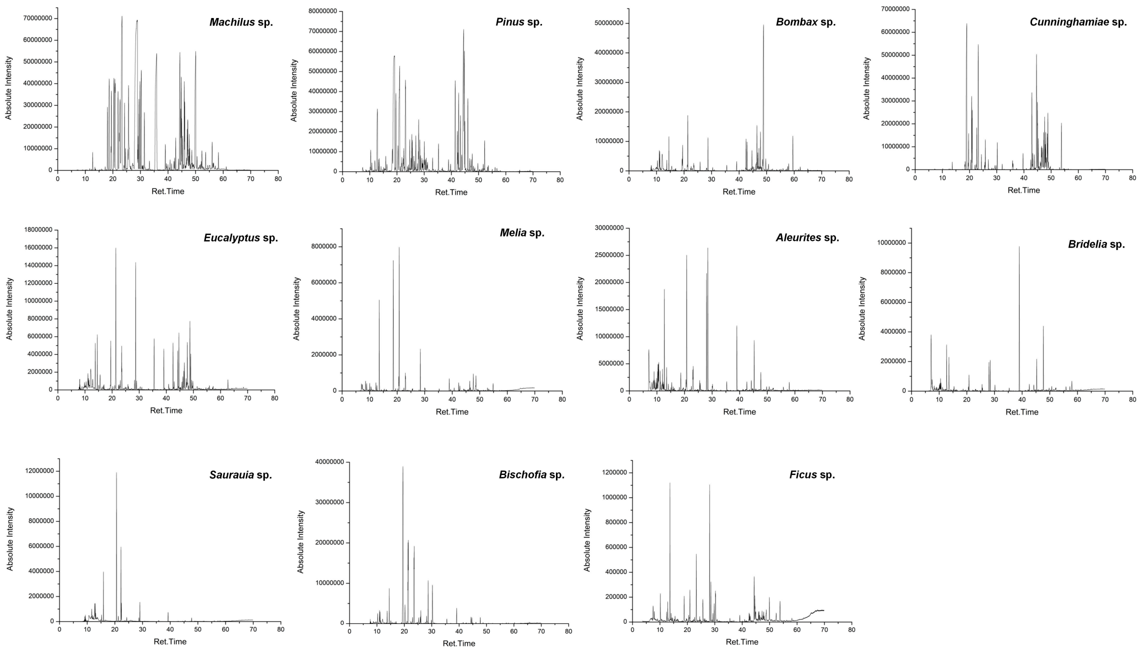

Figure S2. Total Ion Chromatograms of 11 wood species of particleboard raw materials

Table S3. Methods of chemical composition and odor characteristics analysis of odorants in woods

**Sample pretreatment process**	Wood sample (1 g) was added to a 20 mL headspace bottle, which was then capped and sealed. After equilibrating for 30 min at 60 ℃ in the dynamic headspacethermal desorption autosampler (Gerstel, TD3.5 +, Germany), wood samples were purged with a helium stream (ultra-high purity) at 30 mL/min for 20 min. Volatile compounds (including odorants) of the samples were trapped

	into Tenax tubes, then were subjected to GC-MS equipment (Agilent 8890/5977B, American).
**GC-MS test parameters**	Separations in GC were performed on DM-TVOC capillary column (50 m × 0.32 mm × 1 μm), temperature program, 50 ◦C (hold for 2 min) → 120 ◦C (rate 10 ◦C/min, held for10 min) → 160 ◦C (rate 5 ◦C/min, held for 8 min) → 200 ◦C (rate 5 ◦C/min, held for 10 min) → 260 ◦C (rate 5 ◦C/min, held for 5 min), Electron-impact mass spectra were generated at 70 eV, with the m/z scan range being 45–550. The MS source temperature was 240 ◦C. The effluent from the capillary column was split 1:1 v/v between the MS and the olfactometry (Gerstel ODP 3, Germany).
**Compound identification & quantitative analysis using external standard method**	Compounds were identified according to NIST 17.0 mass spectra libraries installed in the GC– MS equipment.Some reagents such as β-pinene (≥95%), α-terpineol (>95%), d-limonene (>95%), fenchol (>96%), hexanal (>99%) were used for further confirmation. Quantitative analysis was carried out by external standard method. In addition, Prepared five different concentrations toluene standard solutions (0.05 μg/μL, 0.1 μg/μL, 0.2 μg/μL, 0.4 μg/μL, 0.5 μg/μL). These standard solutions were introduced into the Tenax sorbent using a 1 μL syringe. The peak areas of toluene chromatogram were proportional to the half mass of toluene injected. The slope of the calibration curve over the linear range was the response factor of the odor compounds studied. Odor compounds were quantified using the calibration response factor for toluene.
**Odor assessments**	Odor assessment was performed by three experienced panelists. By gradually increasing the split ratio of the gas chromatography inlet, the odor assessment team had a 50 % chance of producing an olfactory physiological response. Then, the mass of odorous substances at the olfactory end was divided by the total gas volume per unit time at the olfactometry outlet, and this team average value was defined as the threshold odor concentration (TOC) of odorous substances, unit mg/m3. During the reproducibility of the experimental runs, a±5.0% standard deviation was observed for the TOC value.

Table S4. Quantity of compounds in different wood species of fiberboard raw materials

	C. camphora	*Pinus*	*Ligustrum*	*Salix*	*Fagus*	*Rhaphiolepis*	*Prunus*	*Populus*	*Pterocarya*	*Firmiana*	*Celtis*
terpene	30	15	14	12	14	17	13	12	16	5	4
aldehyde	1	6	4	2	5	4	3	5		1	1
ketone	3	6	2	3	1	2	2	1	1	2	2
alcohol	10	5	4	6	6	1		3		1	2
benzene series		2	5	4	1	5	5		5	2	1
alkane		1	1	1	2			2			
aromatic	1	1	1	1	1	1	2	1	1		

hydrocarbon											
ether		1	3	3	1	1	2	1	1	1	1
acid		1		1	1		1				
ester	1	1									
phenol	2										
SUM	48	39	34	33	32	31	28	25	24	12	11

Table S5. Quantity proportion of compound categories in different wood species of fiberboard raw materials

	C. camphora	*Pinus*	*Ligustrum*	*Salix*	*Fagus*	*Rhaphiolepis*	*Prunus*	*Populus*	*Pterocarya*	*Firmiana*	*Celtis*
Total number of Categorie	7	10	8	9	9	6	7	7	5	6	6
terpene	63%	38%	41%	36%	44%	55%	46%	48%	67%	42%	36%
aldehyde	23%	15%	12%	6%	16%	13%	11%	20%		8%	9%
ketone	63%	15%	6%	9%	3%	6%	7%	4%	47%	17%	18%
alcohol	21%	13%	12%	18%	19%			12%		8%	18%
benzene series		5%	15%	12%	3%	16%	18%		21%	17%	9%
alkane		3%	3%	8%	6%			8%			
aromatic hydrocarbon	2%	3%	3%	3%	3%	3%	7%	4%	4%		
ether		3%	9%	9%	3%	3%	7%	4%	4%	8%	9%
acid				3%	3%		3%				
ester	2%	3%									
phenol	4%										

Table S6. Total concentration of odor substances in different wood species of fiberboard raw materials (Unit: mg/m^3)

	C. camphora	*Pinus*	*Ligustrum*	*Salix*	*Fagus*	*Rhaphiolepis*	*Prunus*	*Populus*	*Pterocarya*	*Firmiana*	*Celtis*
terpene	36.89	38.55	0.12	0.03	0.05	0.11	0.06	0.24	0.07	0.02	0.003
aldehyde	0.02	0.20	0.01	0.004	0.02	0.01	0.01	0.06		0.002	0.003
ketone	26.74	0.26	0.01	0.05	0.24	0.01	0.004	0.001	0.02	0.003	0.002
alcohol	37.39	0.44	0.01	0.01	0.13	0.001		0.02		0.002	0.002
benzene series		0.02	0.01	0.01	0.001	0.02	0.01		0.01	0.002	0.004
alkane		0.01	0.001	0.001	0.01		0.002	0.01			
aromatic hydrocarbon	0.24	0.03	0.002	0.004	0.001	0.003	0.002	0.02	0.002		
ether		0.37	0.004	0.01	0.002	0.004	0.01	0.003	0.001	0.001	0.001

acid		0.03		0.05	0.002		0.02				
ester	0.04	0.02					0.002				
phenol	0.08						0.002				
SUM	101.40	39.93	0.16	0.16	0.46	0.15	0.11	0.35	0.10	0.03	0.02

Table S7. Concentration proportion of odor substances in different compound categories of wood species for fiberboard raw materials

	C. camphora	*Pinus*	*Ligustrum*	*Salix*	*Fagus*	*Rhaphiolepis*	*Prunus*	*Populus*	*Pterocarya*	*Firmiana*	*Celtis*
terpene	36%	97%	74%	18%	11%	71%	52%	68%	68%	62%	20%
aldehyde	0.02%	0.5%	5%	2%	4%	8%	5%	19%		8%	20%
ketone	63%	0.6%	6%	30%	52%	5%	4%	0.2%	20%	12%	13%
alcohol	37%	1%	3%	6%	28%	1%		5%		8%	13%
benzene series		0.1%	8%	4%	0.2%	10%	5%		9%	8%	27%
alkane		0.03%	1%	1%	2%		2%	3%			
aromatic hydrocarbon	0.2%	0.1%	1%	2%	0.2%	2%	2%	4%	2%		
ether		1%	2%	6%	0.4%	3%	5%	1%	1%	4%	7%
acid		0.1%		30%	0.4%		21%				
ester	0.04%	0.1%					2%				
phenol	0.08%						2%				

Table S8. Quantity of compounds in different wood species for particleboard raw materials

	Machilus	*Pinus*	*Saurauia*	*Cunninghamiae*	*Eucalyptus*	*Melia*	*Bombax*	*Aleurites*	*Bridelia*	*Bischofia*	*Ficus*
terpene	61	56	40	90	50	54	76	24	26	50	25
aldehyde	4	18	17		10	8	12	28	30	23	20
ketone	9	5	3		7	4	4	4	9	9	
alcohol	15	18	20	7	23		8	20	9	9	20
benzene series		15	10			12		12	9		20
alkane			7			15		4	9		10
aromatic hydrocarbon	2	3		3	7	4		4	4	5	5
ether	2	3			3	4					
acid								4			
ester	7								4	5	
phenol	61	56	40	90	50	54	76	24	26	50	25

SUM	46	39	30	30	30	26	25	25	23	22	20

Table S9. Quantity proportion of compound categories in different wood species for particleboard raw materials

	Machilus	*Pinus*	*Saurauia*	*Cunninghamiae*	*Eucalyptus*	*Melia*	*Bombax*	*Aleurites*	*Bridelia*	*Bischofia*	*Ficus*
Total number of Categorie	7	7	7	3	6	7	4	8	8	6	6
terpene	61%	56%	40%	90%	50%	54%	76%	24%	26%	50%	25%
aldehyde	4%	18%	17%		10%	8%	12%	28%	30%	23%	20%
ketone	9%	5%	3%		7%	4%	4%	4%	9%	9%	
alcohol	15%	18%	20%	7%	23%		8%	20%	9%	9%	20%
benzene series		15%	10%			12%		12%	9%		20%
alkane			7%			15%		4%	9%		10%
aromatic hydrocarbon	2%	3%		3%	7%	4%		4%	4%	5%	5%
ether	2%	3%			3%	4%					
acid								4%			
ester	7%								4%	5%	
phenol			3%								

Table S10. Total concentration of odor substances in different wood species for particleboard raw materials (Unit: mg/m^3)

	Machilus	*Pinus*	*Saurauia*	*Cunninghamiae*	*Eucalyptus*	*Melia*	*Bombax*	*Aleurites*	*Bridelia*	*Bischofia*	*Ficus*
terpene	29.92	31.49	0.17	8.33	0.08	0.02	2.07	0.001	0.01	0.38	0.004
aldehyde	0.01	0.48	0.06		0.02	0.002	0.01	0.02	0.02	0.02	0.004
ketone	28.11	0.01	0.01		0.01	0.001	0.09	0.01	0.003	0.004	0.001
alcohol	4.001	0.2	0.06	0.04	0.07		0.02	0.02	0.007	0.01	0.01
benzene series			0.01			0.002		0.003	0.002		0.012
alkane			0.002			0.01		0.001	0.001		0.002
aromatic hydrocarbon	0.05	0.01		0.01	0.003	0.001		0.003	0.001	0.002	0.001
ether	0.15	0.04			0.01	0.003					
acid								0.002			
ester	0.08								0.001	0.02	
phenol			0.002								
SUM	62.32	32.23	0.30	8.38	0.19	0.04	2.19	0.06	0.04	0.44	0.03

Table S11. Concentration proportion of odor substances in different compound categories of wood species for particleboard raw materials

	Machilus	*Pinus*	*Saurauia*	*Cunninghamiae*	*Eucalyptus*	*Melia*	*Bombax*	*Aleurites*	*Bridelia*	*Bischofia*	*Ficus*
terpene	48%	98%	56%	99%	43%	56%	95%	2%	17%	87%	12%
aldehyde	0.02%	1%	20%		11%	5%	1%	33%	42%	4%	12%
ketone	45%	0.03%			5%	2%	4%	17%		1%	3%
alcohol	6%	1%	20%	0.4%	37%		1%	33%	19%	3%	29%
benzene series			2%			5%		5%	6%		35%
alkane			1%			23%		2%	3%		6%
aromatic hydrocarbon	0.1%	0.03%		0.1%	2%	2%		5%	3%	0.4%	2%
ether	0.2%	0.1%			3%	7%					
acid			0.002%					3%			
ester	0.1%								2%	5%	
phenol			1%								
